# Tuning social modulations of gaze cueing via contextual factors

**DOI:** 10.3758/s13423-022-02211-z

**Published:** 2022-11-07

**Authors:** Xinyuan Zhang, Mario Dalmaso, Giovanni Galfano, Luigi Castelli

**Affiliations:** 1 Department of Developmental and Social Psychology, University of Padova, 35131 Padova, Italy; 2 School of New Media, Financial & Economic News, Guangdong University of Finance, Guangzhou, 510520 China; 3 Department of Psychology and Center for Brain and Cognitive Sciences, School of Education, Guangzhou University, Guangzhou, 510006 China

**Keywords:** Gaze cueing, Automaticity, Context, Social modulation, Spatial attention

## Abstract

Gaze cueing reflects the tendency to shift attention toward a location cued by the averted gaze of others. This effect does not fulfill criteria for strong automaticity because its magnitude is sensitive to the manipulation of different social features. Recent theoretical perspectives suggest that social modulations of gaze cueing could further critically depend on contextual factors. In this study, we tested this idea, relying on previous evidence showing that Chinese participants are more sensitive to gazes on White than on Asian faces, likely as a consequence of differences in perceived social status. We replicated this effect when we made group membership salient by presenting faces belonging to the different ethnicities in the same block. In contrast, when faces belonging to different ethnicities were presented in separate blocks, a similar gaze-cueing effect was noted, likely because no social comparison processes were activated. These findings are consistent with the idea that social modulations are not rigid but are tuned by contextual factors.

## Introduction

“Gaze cueing” refers to the observation that participants are faster in reacting to targets appearing in locations that a human model previously looked at than to targets appearing in non–gazed-at locations, even if gaze direction is not informative with respect to where the target will appear. This phenomenon is thought to reflect the shift of attention elicited by gaze direction (e.g., Friesen & Kingstone, 1998; see McKay et al., 2021, for a review). An important line of research has attempted to establish whether gaze cueing can be considered an automatic phenomenon (Driver et al., 1999; Friesen et al., 2004; Tipples, 2008). In this regard, automaticity could be related, for instance, to the amount of cognitive resources (automatic processes typically require a negligible pool of resources; e.g., Bobak & Langton, 2015), the awareness of the triggering stimulus (automatic processes typically do not require conscious processing of the stimulus; e.g., Sato et al., 2007), and impermeability to cognitive control (automatic processes are typically impervious to expectancies and resistant to suppression; e.g., Dalmaso et al., 2020b; Galfano et al., 2012; Kuhn & Kingstone, 2009). A different approach to test automaticity focuses on the possibility that gaze cueing can be modulated by social factors. The rationale underlying this approach is that if the gaze-cueing effect is strongly automatic, then it should be resilient to social variables.

Recently, Dalmaso et al. (2020a) reviewed the most relevant social variables that could affect gaze cueing, and identified three different classes of factors: (a) characteristics of the observer (e.g., gender, see Bayliss et al., 2005; Hayward & Ristic, 2017), (b) characteristics of the cueing faces (e.g., dominance and emotional expressions, see Jones et al., 2010, Kuhn & Tipples, 2011; Lassalle & Itier, 2015; McCrackin & Itier, 2018; Ohlsen et al., 2013) and (c) the relationship between the two. This last factor is particularly relevant for the present work. In this regard, several studies have focused on the role of ethnic membership of both the observer and the cueing face by considering different intergroup settings. One set of studies has shown that White participants consistently display an overall larger gaze-cueing effect in response to White than to Black faces (Dalmaso et al., 2015; Pavan et al., 2011; Weisbuch et al., 2017). In contrast, Black participants exhibit a similar gaze-cueing effect irrespective of the ethnicity of the face stimulus (Pavan et al., 2011; Weisbuch et al., 2017). Other studies, addressing Asian and White individuals, have shown that White individuals display a reliable gaze-cueing effect of similar magnitude for both Asian and White faces (Strachan et al., 2017; Zhang et al., 2021a, 2021b). Conversely, Asian participants are more sensitive to the gaze belonging to White than to Asian faces (Zhang et al., 2021a, 2021b). This set of results has been interpreted as reflecting the impact of the different perceived social status associated with the various social groups.

Having established that gaze cueing is not automatic in that it is susceptible to the influence of several social factors, Dalmaso et al. (2020a) proposed a conceptual framework (called “eyeTUNE”) that predicts the possible scenarios under which such social factors may or may not play a modulatory role in the gaze-cueing effect. In the present study, we focused on the key assumption of the eyeTUNE framework, namely that social modulations are not automatic and take place in a rigid and mandatory manner. In contrast, social modulations would crucially depend on overarching factors: situational gain (e.g., whether orienting in response to the perceived gaze cue could lead to any personal benefit), individual constraints (e.g., biological and psychological individual differences), and contextual factors. We focused on the last factor, namely, the broader context in which the (potentially) triggering stimuli are encountered, in the domain of ethnic membership. This perspective is important in that the observed modulations reported in the studies highlighted earlier (e.g., Weisbuch et al., 2017; Zhang et al., 2021a) were likely influenced by the social context that provides meaning to group membership (Dalmaso et al., 2020a). The manipulation of contextual factors can rely on explicit information about the social background (e.g., Chen & Zhao, 2015). Moreover, in an attempt to disentangle between perceptual and social accounts of the asymmetric gaze-cueing effect exhibited by White participants in response to Black and White faces, Pavan et al. (2011) manipulated the saliency of ethnic membership by changing the comparative setting. The results showed that when only Black faces were presented, they triggered a reliable gaze-cueing effect that did not differ in magnitude from that elicited by White faces. The relevance of a comparative setting has also been demonstrated in other domains, such as emotional expressions. For example, Kuhn et al. (2016) manipulated the relative frequency of fearful and happy faces: Fearful faces either appeared more frequently or less frequently than happy faces. In so doing, Kuhn et al. reported a larger gaze-cueing effect for fearful over happy faces only when trials with fearful faces represented rare occurrences – and were therefore more salient – within a context of predominantly happy faces. In contrast, when happy faces were rare, the affective context did not play any modulatory role on gaze cueing, which is in line with the notion that positive emotions are less likely to shape gaze cueing (see, e.g., Dalmaso et al., 2020a).

In this study, we aimed to further test the relevance of contextual factors on gaze cueing and, more specifically, whether manipulating the presence of a comparative context might affect the asymmetric gaze-cueing effect exhibited by Chinese participants in response to Asian and White faces documented in previous research (Zhang et al., 2021a, 2021b). This is particularly important given the peculiar pattern of gaze-cueing effect exhibited by Chinese participants, who oriented their attention more strongly in response to the gaze of White faces (i.e., the outgroup). Hence, we performed an experiment in which Chinese participants were presented with Asian and White faces, either intermixed or in different blocks of trials. In the former condition, we expected to replicate the pattern reported by Zhang et al. (2021a, 2021b). In contrast, when Asian and White faces were blocked, we predicted, consistent with the eyeTUNE framework, a reliable gaze-cueing effect of similar magnitude for faces belonging to both ethnicities. Blocked presentation indeed provides no straightforward comparative setting and should thus make it less likely that faces are categorised as a function of their ethnicity and that category-based information (e.g., stereotypes) is activated (Macrae & Cloutier, 2009; see also Rees et al., 2020; Todd et al., 2021). In such conditions, gaze can act as a default cue for attention. However, when faces associated with different social characteristics are presented in the same block of trials, a comparative context is explicitly provided, and responses to eye gaze are more likely modulated by the nature of the manipulated social characteristics.

## Method

### Participants

Eighty-one Chinese participants (54 females; *M* = 20 years, range = 18–25) from Guangzhou University were recruited. Sample size was predetermined on the basis of previous studies that have addressed ethnicity-based modulations of gaze cueing in Chinese individuals (see Zhang et al., 2021a, 2021b). Participants were randomly assigned to either a mixed (*n* = 41) or a blocked (*n* = 40) condition. We adopted a between-participants design in order to avoid possible carryover effects between the mixed and the blocked condition. Indeed, an initial administration of the mixed condition would inevitably imply the activation of an intergroup comparison that might remain salient in the subsequent blocked condition. Participants had normal or corrected-to-normal sight and received either course credits or ¥10 (~US$1.40) for participating. All of them provided signed informed consent. The study was approved by the institutional review board of the Educational School, Guangzhou University.

### Apparatus and stimuli

The experiment was controlled by E-Prime software on a PC equipped with a 43-cm monitor (1,024 × 768 pixels, 60 Hz). The monitor was placed 57 cm away from the participants. The stimuli were presented on a black background. Sixteen avatar faces, created with FaceGen 3.1 software (https://facegen.com), were used. Eight avatars depicted male individuals (four Asian and four White), whereas the other avatars depicted female individuals (four Asian and four White). The stimuli were the same as in Zhang et al.’s (2021a) study. All pictures were the same size (14.4° wide × 16.8° high). Each face had three different versions: one displaying a direct gaze, one displaying a gaze averted leftward, and the other displaying a gaze averted rightward.

### Design and procedure

**Fig. 1:**
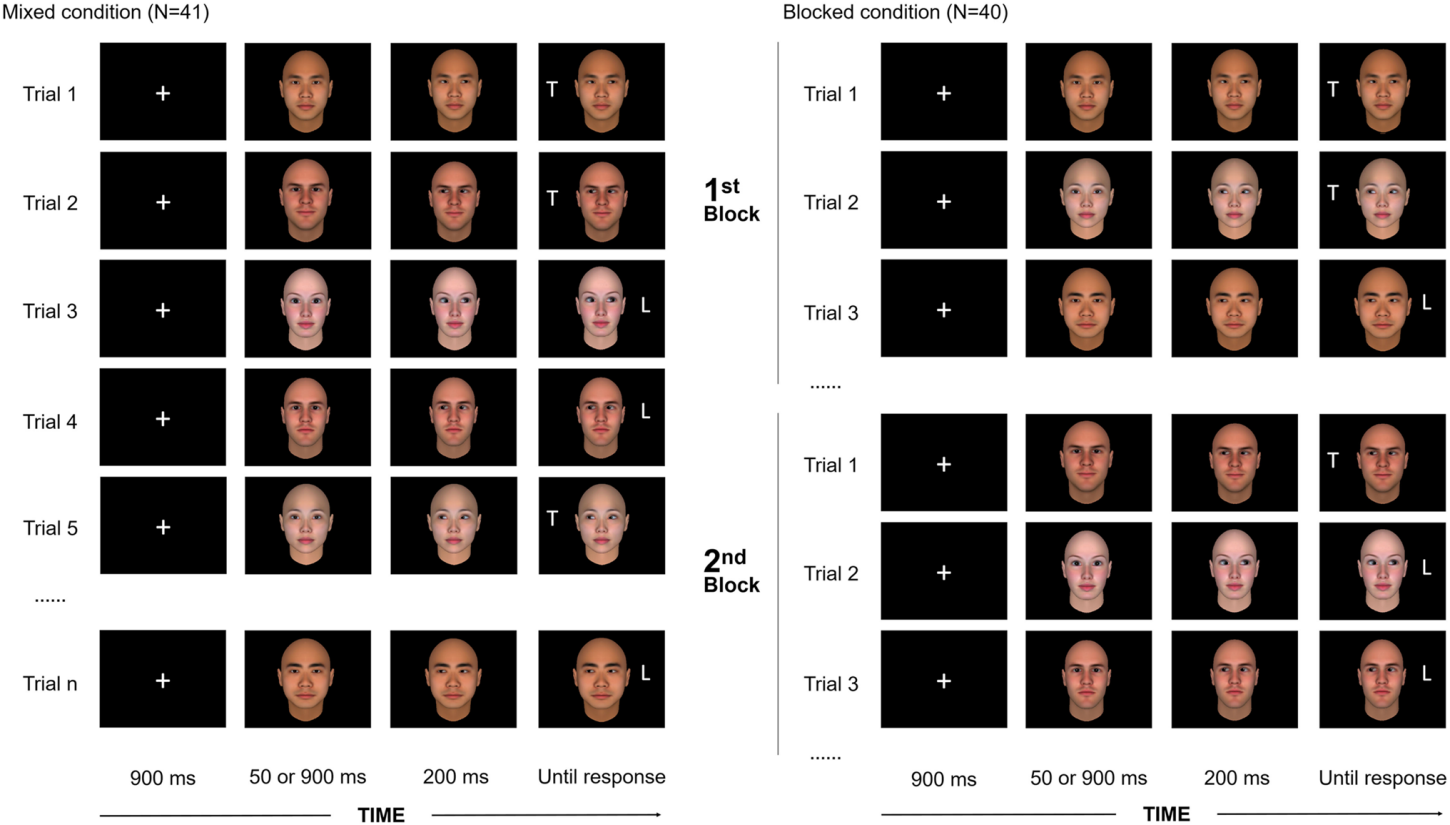
Experimental procedure and examples of stimuli. *Note*. The left panel illustrates the trial sequence in the mixed condition, in which White faces were intermixed with Asian faces and presented in a random manner. The right panel illustrates the trial sequence in the blocked condition, in which White and Asian faces were presented in two different blocks. We counterbalanced the order of the two blocks across participants

In the mixed condition, White faces were intermixed with Asian faces. In the blocked condition, the procedure was the same as in the mixed condition, except that White and Asian faces were presented separately in two blocks, the order of which was counterbalanced across participants. In both conditions, each trial began with a 900-ms white central fixation cross, which was afterward replaced by a direct-gaze face. After either 50 or 900 ms, the same face was presented gazing either leftward or rightward. After 200 ms, a target letter (an L or a T in 24-point Arial bold font) appeared either 11° leftward or rightward with respect to the centre of the screen (see Fig. 1). Temporal parameters of direct-gaze faces (i.e., 50 vs. 900 ms) were the same as in Zhang et al. (2021a; Experiments 3 and 4). This choice was made in order to keep the experimental designs as similar as possible and, although Zhang et al. did not find any modulatory role of temporal parameters, we aimed to further test the occurrence of eventual variations in the time course of the social modulations. Indeed, because social modulations can be sensitive to temporal parameters (e.g., Dalmaso et al., 2014; Jones et al., 2010), participants may rapidly activate social knowledge associated with group membership of the face, but then this knowledge might fade away from working memory because it is not relevant to the task at hand. The target remained on-screen until the participant provided a manual response by pressing either the “D” or the “K” key, depending on the target identity. We counterbalanced the key-target association across participants. Participants were instructed to be both fast and accurate in providing their responses. In spatially congruent trials, the target appeared in the gazed-at location, whereas in spatially incongruent trials the target appeared in the opposite location. Congruent and incongruent trials occurred with the same frequency and were randomly presented within each block. In total, each participant was administered 256 trials^1^ (128 trials in each block of the blocked condition). Participants were instructed that gaze direction was task irrelevant and to maintain fixation at the center of the screen throughout a trial.

## Results

Incorrect responses were removed and analysed separately (5.24% of trials). Reaction times (RTs) for correct responses below or above 3 *SD* from the mean of each participant, for each cell of the design, were also removed (1.38% of trials). We conducted a mixed analysis of variance (ANOVA) on mean RTs, including congruency (congruent vs. incongruent), direct-gaze frame duration (50 vs. 900 ms), and face ethnicity (Asian vs. White), as within-participants factors, and condition (blocked vs. mixed) as a between-participants factor. Bonferroni correction was applied for multiple comparisons.

**Fig. 2:**
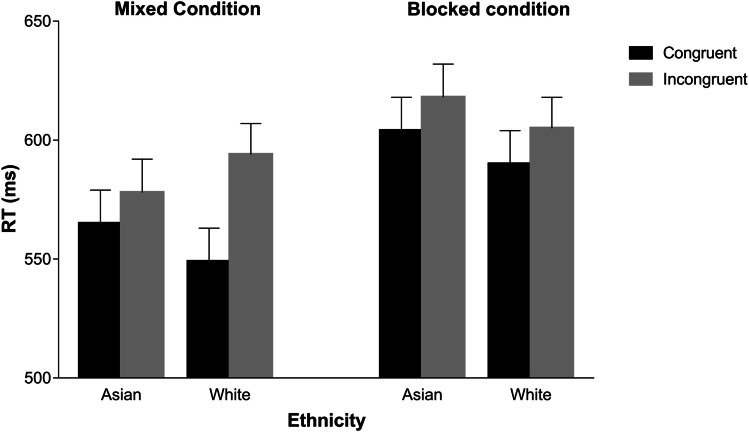
Reaction times (RTs) for correct responses as a function of spatial congruency, ethnicity of the faces, and condition. *Note*. Error bars represent standard errors

A significant gaze-cueing effect emerged, *F*(1, 79) = 78.73, *p* < .001,* η*^2^_p_ = .50, with shorter RTs on congruent (*M* = 577 ms, *SE* = 9.47) than on incongruent trials (*M* = 599 ms, *SE* = 9.40). Direct-gaze frame duration also yielded a significant main effect, *F*(1, 79) = 19.51, *p* < .001,* η*^2^_p_ = .20, reflecting longer RTs for the short (*M* = 595 ms, *SE* = 9.39) than for the long duration (*M* = 580 ms, *SE* = 9.62). The main effects of condition, *F*(1, 79) = 3.11, *p* = .082,* η*^2^_p_ = .04, and face ethnicity, *F*(1, 79) = 2.98, *p* = .088,* η*^2^_p_ = .04, were not significant. A significant Congruency × Face Ethnicity interaction was observed, *F*(1, 79) = 9.33, *p* = .003,* η*^2^_p_ = .11, indicating that the difference between congruent trials and incongruent trials was greater for White (*M* = 30 ms, *SE* = 4.33) than for Asian faces (*M* = 13 ms, *SE* = 3.14), although the effect of congruency was significant for both White, *t*(79) = 7.41, *p* < .001, *d* = 0.96, and Asian faces, *t*(79) = 4.24, *p* < .001, *d* = 0.43. The Congruency × Condition interaction was also significant, *F*(1, 79) = 8.98, *p* = .004,* η*^2^_p_ = .10, reflecting a greater difference between congruent and incongruent trials in the mixed (*M* = 29 ms, *SE* = 4.11) than in the blocked condition (*M* = 14 ms, *SE* = 2.53); the effect of congruency, however, was significant for both the mixed, *t*(79) = 8.45, *p* < .001, *d* = 0.93, and the blocked condition, *t*(79) = 4.13, *p* < .001, *d* = 0.46. Consistent with Zhang et al. (2021a), direct-gaze frame duration was not involved in any significant interaction (*F*s < 1.80, *p*s > .183), thus suggesting that the manipulated temporal parameters did not play any modulatory role on gaze cueing. More important, the Congruency × Face Ethnicity × Condition interaction was significant, *F*(1, 79) = 8.76, *p* = .004 *η*^2^_p_ = .10 (see Fig. 2). No other significant results emerged (*F*s < 2.74, *p*s > .102).^2^ We investigated the meaning of the three-way interaction by running two additional ANOVAs, one for each condition, including two within-participant factors, namely, congruency and ethnicity. As for the mixed condition, the main effect of congruency was significant, *F*(1, 40) = 49.42, *p* < .001,* η*^2^_p_ = .55, with shorter RTs on congruent (*M* = 557 ms, *SE* = 12.70) than on incongruent trials (*M* = 586 ms, *SE* = 12.80). Importantly, the Congruency × Ethnicity interaction was significant, *F*(1, 40) = 11.34, *p* = .002,* η*^2^_p_ = .22, because the difference between congruent and incongruent trials was bigger for White faces (*M* = 45 ms, *SE* = 7.15) than for Asian faces (*M* = 13 ms, *SE* = 5.25). However, gaze cueing was significant for both White, *t*(40) = 6.31, *p* < .001, *d* = 1.70, and Asian faces, *t*(40) = 2.46, *p* = .036, *d* = 0.49. As for the blocked condition, a significant main effect of congruency emerged, *F*(1, 39) = 31.18, *p* < .001,* η*^2^_p_ = .44, reflecting shorter RTs on congruent trials (*M* = 597 ms, *SE* = 14.10) than on incongruent trials (*M* = 612 ms, *SE* = 13.70). The main effect of face ethnicity was not significant, *F*(1, 39) = 3.10, *p* = .086,* η*^2^_p_ = .07. Crucially, the Congruency × Face Ethnicity interaction was not significant, *F*(1, 39) = .04, *p* = .84,* η*^2^_p_ = .00.

We conducted the same omnibus ANOVA on the percentage of incorrect responses. No relevant results emerged in regard to our hypotheses.^3^

### Impact of block order

The analyses including mixed and blocked conditions demonstrated that the presence of an experimental context favouring (or not) stimulus comparison modulated the strength of gaze cueing in response to faces belonging to different ethnicities. Following this logic, we addressed the potential influence of block order in gaze cueing for Asian versus White faces in the blocked condition; specifically, in an exploratory perspective, we assessed whether differences, if any, in the gaze-cueing effect may arise in the second block of trials, that is, after a comparison setting had been established (see Ristic & Kingstone, 2005). To this end, we re-analysed data for the blocked condition including block order (Asian faces first vs. White faces first) as a between-participants factor. A significant Congruency × Ethnicity × Block Order interaction emerged, *F*(1, 38) = 6.80, *p* = .013,* η*^2^_p_ = .15. In the Asian-faces-first condition, congruency yielded a significant main effect, *F*(1, 20) = 20.48, *p* < .001,* η*^2^_p_ = .51, whereas the Congruency × Ethnicity interaction was not significant, *F*(1, 20) = 2.01, *p* = .172,* η*^2^_p_ = .09. In the White-faces-first condition, conversely, the interaction was significant, *F*(1, 18) = 8.70, *p* = .009,* η*^2^_p_ = .33. Two-tailed *t* tests showed that the gaze-cueing effect was significant for White faces, *t*(18) = 5.06, *p* < .001, *d* = 1.16, but not for Asian faces, *t*(18) = 1.25, *p* = .456, *d* = 0.37.

## Discussion

As predicted, gaze-cueing effects of similar magnitude steadily emerged for both Asian and White faces when faces belonging to the different ethnicities were presented in separate blocks of trials. In sharp contrast, the magnitude of gaze cueing significantly changed when Asian and White faces were presented intermixed in the same block of trials, with a significantly smaller effect for Asian faces, in line with previous evidence (Zhang et al., 2021a, 2021b). This pattern suggests that the modulatory effect exerted by ethnicity can be observed provided that faces are presented in an intermixed fashion, thus making their group membership more salient through comparison processes (see also Pavan et al., 2011). Converging evidence emerged from an exploratory analysis for the blocked condition data, in which the impact of block order was examined. Social variables exerted a stronger impact in the second block, namely, after stimuli presented in the first block had likely activated a term of comparison based on social categories (Macrae & Cloutier, 2009).

This study provides further evidence that the link concerning the observer’s characteristics and the features of the face providing the eye gaze cue is critical in modulating gaze cueing. On the one hand, the gaze-cueing effect cannot be said to be strictly automatic because of the robust modulations as a function of social variables documented in the literature (Dalmaso et al., 2020a). On the other hand, however, the occurrence of these modulatory effects is not unconditional in that they do not invariably emerge. In this regard, the eyeTUNE framework (Dalmaso et al., 2020a) posits that modulatory effects are neither rigid nor independent of contextual factors. A systematic investigation into the role of context in gaze cueing is still relatively scarce. Interestingly, the role of context has been mainly associated with different aspects of motivational drives. For instance, affective priming (Ishikawa et al., 2021) and being primed with rejection-related thoughts (Wilkowski et al., 2009) have been shown to enhance gaze-cueing effects. Moreover, a recent study (Dalmaso et al., 2021) provided evidence that social deprivation results in a magnified responsiveness to the gaze direction of others. All these studies share a common definition of context, namely, the observer’s background internal states while performing the task. However, context can also be conceptualised in a different manner. In particular, in addition to manipulations based on motivational (i.e., top-down) drives, bottom-up factors could play a role. For instance, experimental manipulations might simply rely on the specific sequence of presentation of the various faces (i.e., the faces presented before the upcoming face serve as a background context, in a stimulus-driven fashion). As shown in the present study, the ethnic group membership of the faces can modulate gaze cueing depending on whether the social information conveyed by the faces is made salient by the faces presented in the other trials, which provide a term of comparison. Similarly, as Kuhn et al. (2016) showed, specific emotional expressions can modulate gaze cueing when faces conveying other emotional expressions provide a predominant background.

Even if following the gaze of other individuals is a functional process that can facilitate the attainment of one’s goals (Capozzi & Ristic, 2018), it is now well established that gaze does not always act as a default spatial cue for attention and that gaze cueing is subject to modulations as a function of several social variables (Dalmaso et al., 2020a). This, however, does not imply that the gaze belonging to faces with specific physical or social features necessarily always leads to a reduced, null or enhanced gaze cueing. As postulated in the eyeTUNE framework (Dalmaso et al., 2020a), social modulations critically depend on contextual variables. Indeed, by definition, the meaning of social information is shaped by the social environment in which it is embedded and, at the same time, the social environment can emphasise the relevance of some features of a social stimulus while minimising others.

The present results provide support for the notion that social modulations of gaze cueing can be tuned by means of contextual factors. As in typical situations of our daily life, the tendency to follow the eyes of other individuals appears to fluctuate as a function of the mutable contexts in which we encounter other people.

## Footnotes

1 This results from the combination of the following variables: gaze direction (2) **×** face ethnicity (2) **×** target location (2) **×** target identity (2) **×** direct-gaze frame duration (2) **×** face identity (8).

2 Because no a priori power analysis was conducted, we followed the approach suggested by Dziak et al. (2020) and performed Bayesian model selection analyses. This a posteriori method revealed that the statistical model that best accounted for the observed data included the crucial triple interaction involving congruency, face ethnicity, and condition. In particular, there was moderate evidence favouring the model including the triple interaction over the best fitting model that did not include it (Bayes factor = 6.25; see also Wagenmakers et al., 2018).

3 Only a significant main effect of direct-gaze frame duration emerged, *F*(1, 79) = 5.42, *p* = .023,* η*^2^_p_ = .06, reflecting a higher percentage of incorrect responses for the long (*M* = 5.58%, *SE* = 0.42%) than for the short (*M* = 4.90%, *SE* = 0.37%) duration. Congruency, *F*(1, 79) = 1.88, *p* = .175, *η*^2^_p_ = .02; face ethnicity, *F*(1, 79) = 2.75, *p* = .101,* η*^2^_p_ = .03; and condition, *F*(1, 79) = 0.40, *p* = .529, *η*^2^_p_ = .01, did not yield significant effects. The same held true for the interactions (*F*s < 1.27, *p*s > .262).
